# People, planet and participation: the Kuching statement on healthy, just and sustainable urban development

**DOI:** 10.1093/heapro/daw046

**Published:** 2016-06-29

**Authors:** International Institute for Global Health, United Nations University, Kuala Lumpur, Malaysia

**Keywords:** sustainable development goals, determinants of health, urban health

## Abstract

This statement was commissioned by the UNU International Institute for Global Health in the run up to Habitat III—the third United Nations conference on housing and sustainable urban development. The statement draws on insights from the World Urban Campaign thinkers campus held during 24–27 January 2016 in Kuching, a WHO-designated healthy city.

## INTRODUCTION

We are entering the Anthropocene—a new geological time period that marks an age of colossal and rapid human impact on Earth’s systems. In this rapidly urbanizing era, cities are key players in ensuring that humanity and all other species can live harmoniously and healthily on this one small planet. But this requires cities to adopt an eco-social approach, placing both the health of people and planet at the centre of urban planning and governance. The hallmark of successful 21st century cities will be an understanding of urban development in terms of the complex interconnections between the ecological, economic and social foundations of human development and health.

## Planet: cities living within ecological boundaries

We disrupt and destroy Earth’s systems at our own peril. Humans share this planet with many other species, bounded within the diverse ecosystems that sustain us. People cannot thrive without the Earth’s support systems or the biodiversity of natural ecosystems. Sustainable cities need to operate within the confines of our Earth’s carrying capacity, recognizing that the fundamental roots of human wellbeing—air, water, food, fuels and materials—are derived from natural systems and the ecosystem services they provide. We must value the planet in its role as provider and protector; moderating and regulating climate, recycling and detoxifying wastes, replenishing and restoring the air we breathe and the water we drink, and shielding us from ultra-violet radiation.

In recognizing the limits of the Earth’s natural systems, healthy, just and sustainable cities seek to minimize their ecological footprint whilst maximizing their level of human development. They embrace ecologically sustainable technologies and design and encourage social, cultural and economic activities that protect and enrich their environments without compromising local and remote ecosystems, now and into the future.

## People: cities focused on health and wellbeing

People’s physical, mental and social wellbeing is the core business of cities. While higher levels of government tend to focus first on the economy in measuring progress, successful cities put the focus on quality of life. They understand that the city and its people benefit when all enjoy a high level of human development and health. Urban slums and informal settlements need to be understood not only as inhumane, but as wasteful of human potential.

Healthy cities recognize that they must be socially inclusive as well as ecologically sustainable, and that this requires equity in health and in access to the determinants of health. Creating a healthy and just city starts with securing everyone’s basic human needs for clean air and fresh water, access to fuel and nourishing food, good quality housing, green spaces, education and healthcare in places that are safe and secure.

Only once these basic needs are met can a city begin to unleash the potential of its population. To do so, healthy cities encourage and support innovation, creativity and lifelong education in all their citizens.

Healthy cities support physical activity, mental wellbeing and social connections. They do so by creating environments that are safe, clean and beautiful; restoring areas of natural and cultural heritage; creating great public places; and enabling active transportation, mobility, accessibility and contact with nature. They recognize that humans are social animals, craving comfort, security, conviviality, variety, stimulation and opportunity. They support families and communities, emphasizing respect for diversity and the dignity of others, caring and mutual support, empathy and harmonious relationships.

Socially sustainable cities create opportunities for economic participation for all their citizens, prioritizing decent, safe and stable work that produces goods and services that are themselves health-promoting rather than health-damaging. They also value and support the informal sector, the emerging sharing economy and the unpaid effort of people involved in voluntary work and other ways of contributing to the welfare of the community. Healthy and sustainable cities encourage cultural expression and creative artistic endeavour.

## Participation: Cities adopt governance for health

In order to put people and the planet at the heart of governance, healthy, just and sustainable cities engage fully with their citizens and community organizations. They foster democratic engagement and the active participation of their citizens in the process of decision-making, using the range of participatory processes and technologies the 21st century has to offer.

They create participatory structures and processes to find and embrace common purpose and to manage the affairs of the city in an open and transparent manner. They bring together citizens, public, private and non-profit institutions, business, labour, faith and cultural organizations and other key sectors. They recognize that one sector alone cannot bring forth the vision for healthy people and a healthy planet. Successful cities think laterally and act creatively at multiple-levels and across disciplines. In so doing, they foster resilience.

Healthy cities measure what matters—socially just and ecologically sustainable human development and wellbeing—and use those measures to guide and manage their development. They also ensure that these measures and the monitoring of change are publicly and widely available.

## New tools and approaches

Informed by an understanding of their history, cities of the Anthropocene understand the journey ahead requires new tools and approaches. Past ways of working—characterized by simplistic, linear and siloed approaches that separate cultural, social, economic and ecological dimensions—do not work and indeed make things worse. We need an eco-social approach and sophisticated responses rooted in an understanding that cities are complex systems involving people/people, people/built environment and people/planet interactions. Indigenous and local knowledge is integral to these approaches along with scientific and technical knowledge, as is the use of tools for systems thinking and participatory community education.

Progressive cities recognize the purpose of the economy is to serve the health and welfare of people and planet. Conventional approaches to economic growth characterized by income inequalities and an unsustainable dependence on fossil fuels and other natural resources are questioned, and considered part of the problem. We need to adopt new economic models that seek to maximize natural and social capital and human development, using incentives to encourage the creation of healthy products, services and infrastructure in ecologically sustainable and socially equitable ways. These cities value and protect the livelihoods of informal and vulnerable workers, prioritizing local, self-reliant approaches to economic development while avoiding creating conditions of vulnerability in the first place.

Local creative arts and technology are considered important city assets, tools for change and innovation as well as ways to weave together people and communities both within and across cities. In times of rapid transition or instability they promote health and resilience among city dwellers, providing support to undertake the seemingly momentous and urgent tasks ahead.

By adopting these approaches—by putting people, planet and participation at the heart of governance—cities can lead the way to ecologically sustainable and socially just human development and health.

